# Hydroclimatic adaptation critical to the resilience of tropical forests

**DOI:** 10.1111/gcb.16115

**Published:** 2022-02-20

**Authors:** Chandrakant Singh, Ruud van der Ent, Lan Wang‐Erlandsson, Ingo Fetzer

**Affiliations:** ^1^ 7675 Stockholm Resilience Centre Stockholm University Stockholm Sweden; ^2^ 7675 Bolin Centre for Climate Research Stockholm University Stockholm Sweden; ^3^ Department of Water Management Faculty of Civil Engineering and Geosciences Delft University of Technology Delft The Netherlands; ^4^ Department of Physical Geography Faculty of Geosciences Utrecht University Utrecht The Netherlands

**Keywords:** alternative stable states, ecosystem change, forest‐savanna transition, remote sensing, spatio‐temporal approach, subsoil adaptation, transient state

## Abstract

Forest and savanna ecosystems naturally exist as alternative stable states. The maximum capacity of these ecosystems to absorb perturbations without transitioning to the other alternative stable state is referred to as ‘resilience’. Previous studies have determined the resilience of terrestrial ecosystems to hydroclimatic changes predominantly based on space‐for‐time substitution. This substitution assumes that the contemporary spatial frequency distribution of ecosystems’ tree cover structure holds across time. However, this assumption is problematic since ecosystem adaptation over time is ignored. Here we empirically study tropical forests’ stability and hydroclimatic adaptation dynamics by examining remotely sensed tree cover change (*Δ*TC; aboveground ecosystem structural change) and root zone storage capacity (*S*_r_; buffer capacity towards water‐stress) over the last two decades. We find that ecosystems at high (>75%) and low (<10%) tree cover adapt by instigating considerable subsoil investment, and therefore experience limited *Δ*TC—signifying stability. In contrast, unstable ecosystems at intermediate (30%–60%) tree cover are unable to exploit the same level of adaptation as stable ecosystems, thus showing considerable *Δ*TC. Ignoring this adaptive mechanism can underestimate the resilience of the forest ecosystems, which we find is largely underestimated in the case of the Congo rainforests. The results from this study emphasise the importance of the ecosystem's temporal dynamics and adaptation in inferring and assessing the risk of forest‐savannah transitions under rapid hydroclimatic change.

## Graphical abstract

With the availability of longer time series of remotely sensed datasets, our study tries to incorporate far overlooked temporal support in determining the transient ecosystems’ state and resilience across time. Here, we use the last 20 years of remote sensing data to provide empirical (spatio‐temporal) evidence to alternative stable states and the influence of hydroclimatic adaptation dynamics in maintaining these transient states. Our results also show that ignoring this adaptive capacity can largely underestimate the resilience of the forest ecosystems. Moreover, spatially explicit mapping of these transient states can help strengthen conservation and management efforts.

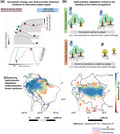

## 1 INTRODUCTION

**FIGURE 1:**
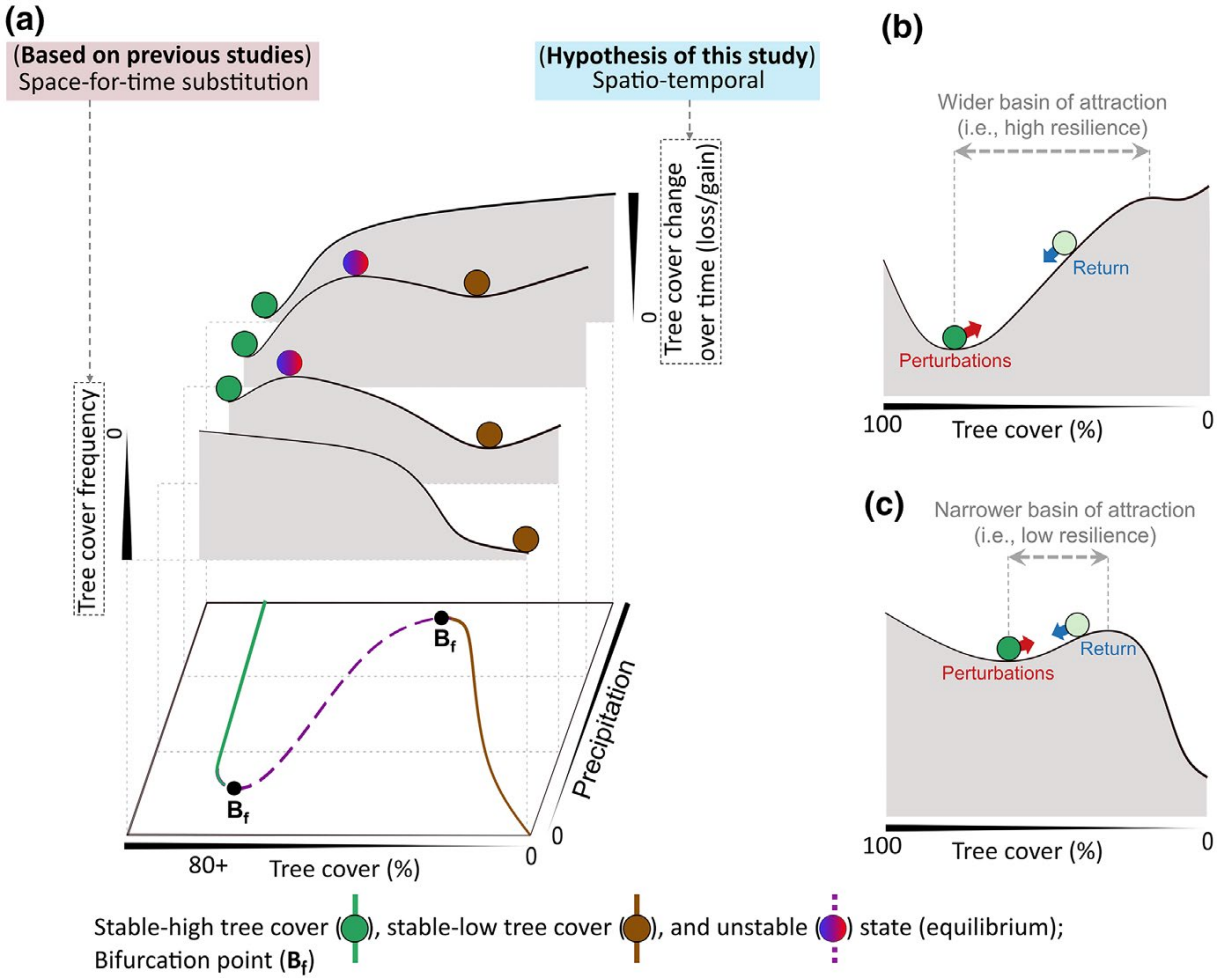
Stability landscape of ecosystems across different mean precipitation (mm year^−1^). (a) The landscape is, originally, based on the frequency distribution of the tree cover [space‐for‐time assumption (Dantas et al., 2016; Hirota et al., 2011; Staver et al., 2011a, 2011b)]. This study substitutes ‘tree cover frequency’ with magnitudes of ‘tree cover change over time’ (spatio‐temporal) for South America and Africa across different classes of precipitation, which we hypothesise should resemble the original landscape. Stable and unstable states (i.e. equilibria) correspond to the valleys (i.e. local minima) and hilltops (i.e. local maxima) in the stability landscapes, respectively. (b, c) The resilience of an individual ecosystem across the stability landscape is represented as the width of the basin of the attraction around a stable state, which declines towards the bifurcation points (i.e. a point where stable and unstable states collide; depicted in (a). Perturbations push the ecosystem towards the hilltop, whereas the ecosystem returns to its stable state when these perturbations are released

Climate change and deforestation reduces the resilience of rainforest ecosystems (Hirota et al., 2011; van Nes et al., 2016), and thus compromise their capacity to remain forests despite various perturbations (Davidson et al., 2012; Malhi et al., 2008). Resilience is quantified and analysed by constructing a ‘stability landscape’, in which valleys (‘basins of attraction’) represent ‘stable states’ and hilltops represent ‘unstable states’ under transition (Figure 1). Resilience is then measured as the width of the basin of attraction around a stable state, which erodes towards bifurcation points (i.e. a point where stable and unstable states collide, becoming unstable) (Hirota et al., 2011; van Nes et al., 2016) (Figure 1a). Within a basin of attraction, stabilising feedbacks help the ecosystem retain its structural and functional characteristics against perturbations (Holling, 1973). The ecosystem will eventually return to its native stable state (‘minimum’ of the basin) when perturbations on the system are released (Figure 1b,c). Beyond a basin of attraction, however, i.e. trespassing a threshold (‘maximum’ of the basin), self‐amplifying feedbacks will instead propel the ecosystem to an alternative stable state (Hirota et al., 2011; Holling, 1973). Therefore, a better understanding of stability and resilience is helpful to evaluate the potential of ecosystem adaptation and systemic risks under future (climatological or non‐climatological) modifications to their conditions (Anderegg et al., 2020).

Due to the lack of analysis of dynamics through time series (Cole et al., 2014; Damgaard, 2019), our present understanding of the stability landscape of the tropical terrestrial ecosystems is based on the frequency distribution of tree cover (Dantas et al., 2016; Hirota et al., 2011; Staver et al., 2011a, 2011b), essentially making a space‐for‐time assumption (Figure 1a). According to this assumption, the frequency distribution determines the size (i.e., width and depth) of the basin of attraction in the conceptual stability landscape, which is then interpreted to be ecosystems stability (deep basin, more stable and vice versa) and resilience (wider basin, more resilient and vice versa) across time (Scheffer et al., 2009) (Figure 1a). However, temporal support to such ecosystem dynamics has not been investigated previously. The availability of longer time series of remote sensing data now allows for a better representation of these ecological states and resilience across time (Damgaard, 2019; Reyer et al., 2015; Singh et al., 2022).

Here, for the first time, to our knowledge, a time series of remotely sensed tree cover change (*Δ*TC) spanning two decades is analysed to investigate rainforest stability and resilience. It is well recognised that the ecosystem's response towards any perturbations is captured in the transient state of the ecosystem (Heimann & Reichstein, 2008; Turner et al., 2003; Wieczynski et al., 2019). Based on this, we hypothesise that the transient state of the ecosystem should resemble the stability landscape found by the space‐for‐time assumption (Figure 1a). Thus, a highly resilient ecosystem will not show considerable *Δ*TC over time, whereas a lowly resilient ecosystem will.

Our hypothesis suggests a correlation between *Δ*TC and the resilience of the ecosystems. However, previous research overlooks any such correlation and only considers the hydroclimate—specifically mean precipitation $\left(\bar{P}\right)$—when quantifying forest resilience (Hirota et al., 2011; Staal et al., 2018). Recent insights, however, hint towards the necessity to also incorporate the buffering capacity of the forest ecosystems, an aspect that is often lacking when representing the ecohydrology of tropical terrestrial ecosystems (van Oorschot et al., 2021; Reyer et al., 2015; Singh et al., 2020). By including root zone storage capacity (*S*_r_), we account for the buffering capacity of the ecosystem in quantifying resilience. *S*_r_ represents the maximum amount of subsoil moisture available to the ecosystems to buffer water deficit during dry periods (Wang‐Erlandsson et al., 2016). This aspect acknowledges that ecosystems respond to water stress (defined here as a deficit in soil water availability inhibiting plant growth) by actively investing in their above‐ and belowground structures to maximise their hydrological benefits (Migliavacca et al., 2021; Singh et al., 2020). Thus, the resulting resilience metric, by also explicitly considering the ecosystems’ adaptive and buffering strategies, should be consistent with actual *Δ*TC.

## 2 METHODS

### 2.1 Study area

This paper focuses on the tropical terrestrial ecosystems of South America and Africa, but the whole study area is slightly larger: 12°N–50°S for South America and 20°N–35°S for Africa. We have used a global administrative database from the Food and Agriculture Organisation (FAO; http://www.fao.org/geonetwork/) to define geographical boundaries for each country and do not have any political intentions behind our research.

### 2.2 Data

We used remotely sensed gauge‐corrected precipitation and evaporation data for our analysis. The daily estimates of precipitation were obtained from the Climate Hazards Group InfraRed Precipitation with Station data (CHIRPS) (Funk et al., 2015) at 0.05° spatial resolution for the years 2000–2019. Furthermore, evaporation in this paper is defined as the sum of all evaporative moisture from the soil and terrestrial vegetation, including those from interception (Miralles et al., 2020). The evaporation datasets chosen for this study were free from any prior assumptions related to biome‐dependent parameterisation (such as plant function types, stomatal conductance, maximum root allocation depth) and soil layer depth (represents maximum depth of moisture uptake) to avoid any artificially introduced transitions between different biomes. Furthermore, these datasets were either derived or validated from actual evaporation estimates (e.g. FLUXNET sites). These conditions narrowed our prospect of using the widely available evaporation datasets. Nevertheless, we created an equally‐weighted ensemble of evaporation using three datasets: (i) Breathing Earth System Simulator (BESS) (Jiang & Ryu, 2016) (ii) Penman‐Monteith‐Leuning (PML) (Zhang et al., 2016) and (iii) FLUXCOM‐RS (Jung et al., 2019). Whilst i and ii were obtained at 0.5°, iii was obtained at 0.083° spatial resolution. All three evaporation datasets were obtained at a monthly timescale for the years 2001–2012. We downscaled these datasets from monthly to daily timescale using the daily estimate of the ERA5 (Hersbach et al., 2020) evaporation at 0.25° spatial resolution.

The aboveground structure of the ecosystem was analysed using the remotely‐sensed MOD44B (version 6) annual tree cover (TC) dataset (Dimiceli et al., 2017) at a fine resolution of 250 m × 250 m for the years 2000–2019. Here, a TC value of 50% would represent a ground coverage of 50% by the canopy in the whole pixel. Furthermore, to minimise the human influence on this analysis, we removed the pixels with human‐influenced land use and non‐terrestrial land cover using the European Space Agency's (ESA) Globcover land‐use classification at 300 m resolution. Ultimately, all the mentioned above datasets were spatially interpolated to 250 m using bilinear interpolation, except for the land‐use dataset which was interpolated using nearest‐neighbour interpolation.

### 2.3 Spatio‐temporal approach for determining ecosystem states

**FIGURE 2:**
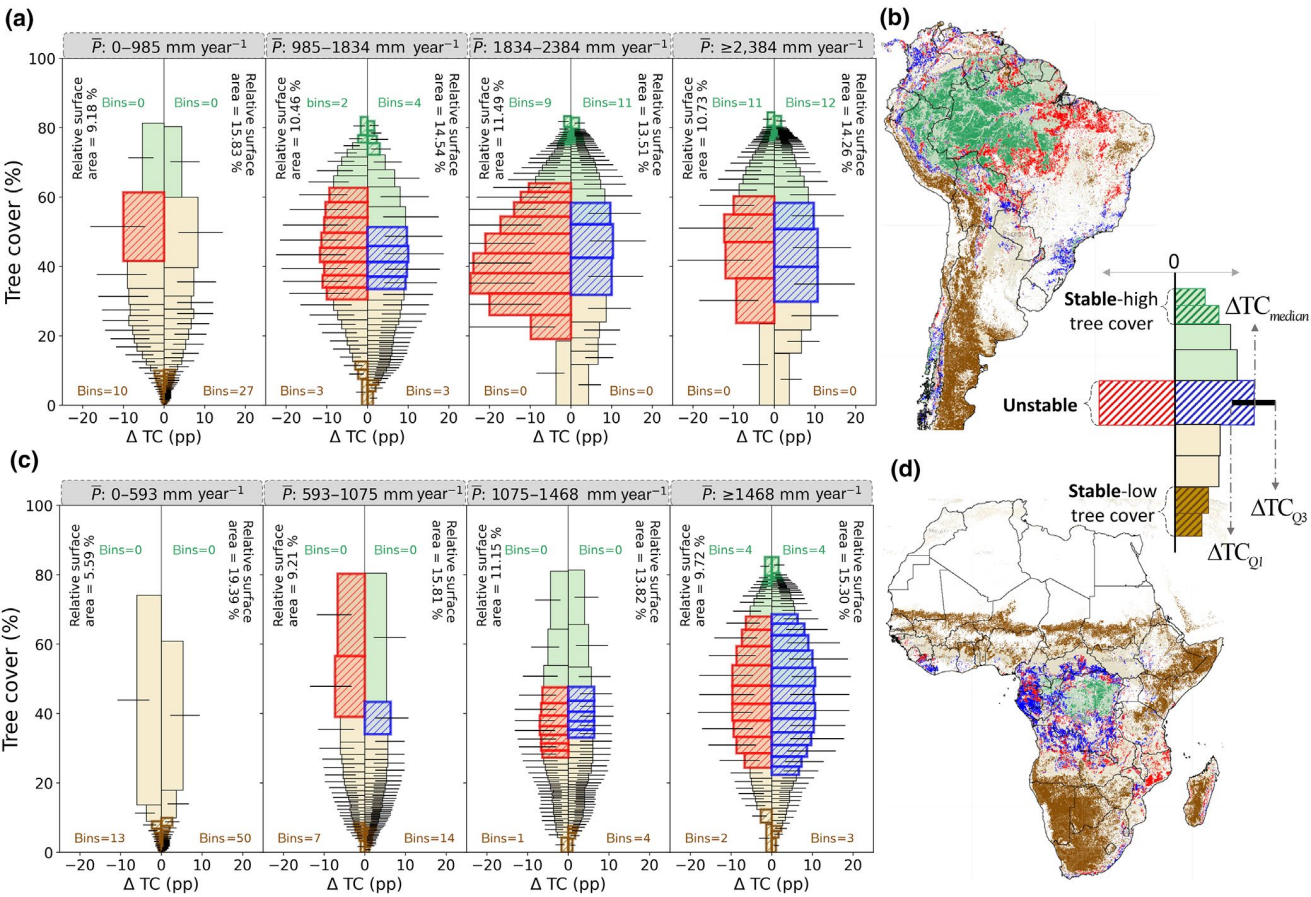
Determining the stability landscape of the tropical terrestrial ecosystem. The landscape for (a, b) South America and (c, d) Africa are analysed using the tree cover (%), tree cover change (*Δ*TC; per cent point [pp]) over the last two decades (from 2000 to 2019; see Section 2), and mean precipitation ($\bar{P}$ [mm year^−1^]; from 2000 to 2019). The total samples are equally divided into four $\bar{P}$ classes. Here, each individual bin within each $\bar{P}$ class corresponds to 2500 pixels. From all these bins, the one with the least *Δ*TC is considered stable, and the one with the most *Δ*TC is unstable (see Section 2). These stable bins at low (dark brown) and high (dark green) tree cover in (a, c) are spatially plotted in (b, d). The unstable bins on either side of *Δ*TC = 0 correspond to tree cover loss (red) or gain (blue). The relative surface area in (a, c) represents the portion of total sample surface area (separately for South America and Africa) on either side of the *Δ*TC = 0. The tree cover extent of stable and unstable bins in (a, c) are spatially plotted in (b, d). The white regions in (b, d) correspond to excluded land cover (Figure S2d). High‐resolution maps of (b, d) can also be visualised at the Google Earth Engine (https://chandrakant.users.earthengine.app/view/state‐of‐ecosystem)

For evaluating these stable and unstable states, a sample size (*n*) of 1,000,000 pixels each—from both continents—from all the 250 m × 250 m pixels was chosen and analysed separately for South America and Africa. This sample was used to determine the tree cover change (*Δ*TC) in the ecosystem structure in the last two decades as follows:

(1) $$\Delta \text{TC =}\;{\bar{\text{TC}}}_{2017‐2019}‐{\bar{\text{TC}}}_{2000‐2002}$$

where ${\bar{\text{TC}}}_{2017‐2019}$ and ${\bar{\text{TC}}}_{2000‐2002}$ represent the mean of the tree cover for the years 2017–2019 and 2000–2002, respectively. Then, we classified the sample (*n*) into four classes based on mean precipitation ($\bar{P}$; Figure 2a,c), with each $\bar{P}$ class representing 25% of the total land area. We further separated each of these $\bar{P}$ classes into tree cover gain (i.e., *Δ*TC ≥ 0) and tree cover loss (*Δ*TC < 0) (Figure 2a,c).

After classifying, we grouped the samples into separate bins sorted by mean tree cover (i.e. ${\bar{\text{TC}}}_{2017‐2019}$; Figure 2a,c), such that each bin represented an equal area (i.e. 2500 sampled pixels = 156.25 km^2^). Lastly, to relate stable and unstable states with the ecosystem's structural change, the 13.4% of the bins with the highest change (i.e. highest *Δ*TC_median_) from all the classes combined were categorised as unstable. Moreover, 38.2% of the bins with the lowest change (i.e. lowest *Δ*TC_median_) were classified as stable. The justification behind selecting the % of stable and unstable bins was based on the area under the distribution curve (Figure S1).

These stable and unstable bins, which were analysed separately for tree cover gain and loss pixels at each $\bar{P}$ class, were plotted spatially at 250 m resolution (Figure 2). For example, our sample analysis suggests that the unstable bins for tree cover loss in South America at $\bar{P}$ class of 0–985 mm year^−1^ falls approximately between 40% and 60% ${\bar{\text{TC}}}_{2017‐2019}$ (Figure 2a). This will be spatially plotted in reality (population) for all the pixels falling between 40% and 60% ${\bar{\text{TC}}}_{2017‐2019}$ at $\bar{P}$ of 0–985 mm year^−1^ for the pixels where *Δ*TC < 0.

### 2.4 Root zone storage capacity

For our analysis, we have considered root zone storage capacity (*S*_r_; derived from daily precipitation and evaporation data) to represent the adaptive buffer capacity of the ecosystem to absorb and adapt to water‐stress conditions. *S*_r_ is the maximum amount of available subsurface moisture that vegetation can store and utilise through their roots for transpiration during dry periods (i.e. periods in which evaporation is greater than precipitation, irrespective of the seasons) (Gao et al., 2014; Wang‐Erlandsson et al., 2016). Plants can increase their S_r_ by expanding their roots in the soil laterally as well as vertically. We adopted the mass‐balance approach by Singh et al. (2020) to derive *S*_r_ from precipitation and evaporation estimates (Supplementary Method 1 in Supplementary Information). The underlying assumption of this approach is that ecosystems would not invest in expanding their storage capacity more than necessary to bridge the water‐deficits it experiences (Wang‐Erlandsson et al., 2016).

### 2.5 Forest resilience and validation

We adapted Hirota et al. (2011) methodology for determining resilience using logistic regressions (Supplementary Method 3 in Supplementary Information). The logistic regression predicts the probability of forest (tree cover >50%) as a function of the independent variable. Hirota et al. (2011) had only considered $\bar{P}$ as the independent variable. However, the new resilience metric proposed in this study also considered *S*_r_ as an independent variable representing the drought buffer capacity of the forest ecosystems. Here, we experimented with $\bar{P}$ and *S*_r_ independently and its combination, and chose the best performing model to represent the ecosystem state (Table S1). We modified the *S*_r_ values for all the regions with tree cover <30% to 99th percentile of each continent's *S*_r_. This is because we assume that forest ecosystems will maximise their storage capacity (i.e. maximise *S*_r_) before transitioning to a savannah (Singh et al., 2020). Lastly, we validate the resilience estimates of $\bar{P}$ and $\bar{P}+S_{r}$ combination for both tree cover loss and gain samples separately against observed *Δ*TC, and assess their performance using spearman rank correlation. A high positive or negative spearman correlation would indicate a high strength and consistency between the resilience estimates and *Δ*TC.

## 3 RESULTS AND DISCUSSION

### 3.1 Tree cover change in relation to stability equilibria

Our spatio‐temporal analysis consistently shows low *Δ*TC for ecosystems at both high (>75%) and low (<10%) tree cover, whereas high *Δ*TC is observed for ecosystems at intermediate (30%–60%) tree cover (Figure 2a,c). A low *Δ*TC for both high and low tree cover ecosystems can be the result of either a minimal perturbation on the ecosystem over the last two decades (2000–2019), or a robust adaptive mechanism that is able to offset the experienced perturbations without considerable change in the ecosystem structure (Singh et al., 2020), which we, therefore, perceive as ‘stable’. Conversely, a high *Δ*TC at intermediate tree cover (Figure 2a,c) implies that the ecosystems in these ranges have been potentially influenced by either strong perturbations (Sutherland et al., 2018) (e.g. deforestation) causing significant changes to their ecosystem structure, or the adaptive mechanism under hydroclimatic changes has modified the ecosystem structure to utilise available resources efficiently. Since we exclude human influences, these high *Δ*TC can solely be explained by tree mortality under climate‐induced water and fire stress (van Nes et al., 2018; Staver et al., 2011a) or tree growth under the influence of wetter climate (Holmgren et al., 2006), thus resulting in these ecosystems undergoing the observed regime shift (Hirota et al., 2011; Scheffer et al., 2009). The self‐amplifying feedback between forest and climate also leads to considerable changes to ecosystem structure, such that forest loss facilitates dry conditions, and dry conditions further influence forest mortality (Staal et al., 2020; Zemp et al., 2017). Overall, structural changes to these ecosystems are much steeper than what we observed for ecosystems in their stable states (Figure 2a,c). Thus, we consider such ecosystems as ‘unstable’. These spatio‐temporal patterns against different $\bar{P}$ levels (Figure 2a,c) further strengthen the presence of stability and instability in terrestrial ecosystems, which previous studies observed using a space‐for‐time assumption (Dantas et al., 2016; Hirota et al., 2011; Staver et al., 2011a, 2011b), can also manifest as actual *Δ*TC over time across the broader tree cover structures.

A closer look at these stable states (i.e. stable‐low and ‐high tree cover bins representing a series of numerical ranges highlighted in dark brown and green, respectively, in Figure 2a,c and spatially highlighted in Figure 2b,d) reveals certain dissimilarities across the $\bar{P}$ classes. Stable‐high tree cover bins decrease gradually with decreasing $\bar{P}$ (Figure 2a,c), thereby implying the inability of the forest ecosystems to maintain their dense structural characteristics under drier conditions (Singh et al., 2020). Here, an increase in *Δ*TC with decreasing $\bar{P}$ suggests that these ecosystems are undergoing a shift to a savannah‐like open‐canopy structure due to intensifying water and fire stress (Hirota et al., 2011; Moser et al., 2010; Zemp et al., 2017). Reversely, stable‐low tree cover bins decrease with increasing $\bar{P}$ (Figure 2a,c). Here, an increase in wetter conditions in the ecosystem helps suppress a fire, thereby preventing fire‐driven seedling mortality (Moser et al., 2010), and drives more soil water storage under a wetter climate (Guan et al., 2015). All these factors thus help promote forest growth and colonisation (Hirota et al., 2011; Uriarte et al., 2018). Nevertheless, the shifting potential, in both these cases, generally manifests itself as a relatively high *Δ*TC within the stable extent (e.g. relatively high *Δ*TC for $\bar{P}$ < 985 mm year^−1^ for South America and $\bar{P}$ < 1468 mm year^−1^ for Africa at a tree cover >70% in Figure 2), with some exceptions (Figure S3).

Interestingly, we also observe that for most of the $\bar{P}$ classes, the extent of the unstable bins (i.e. ranges highlighted in red and blue in Figure 2a,c) is almost similar for both tree cover loss and gain segments. In contrast to stable states, higher potential—suggesting amplified feedback—for both tree cover loss and gain at intermediate tree cover was already hypothesised in a space‐for‐time based approach (Hirota et al., 2011) (Figure 1a) and is confirmed by observable evidence at field scale. For example, open forest structure is promoted under increasingly drier conditions (McAlpine et al., 2018), the influence of fire (Pivello et al., 2021) and fragmentation (Nikonovas et al., 2020). Whereas, forest growth is promoted under El Niño‐southern oscillation influenced wet conditions (Gutiérrez et al., 2008; Holmgren et al., 2006), sustainable management (Chazdon et al., 2020; Lewis et al., 2019; Wilson et al., 2019) and conservation efforts undertaken by local authorities to reduce deforestation, extensive grazing and wildfires (Cheung et al., 2010; Guedes Pinto & Voivodic, 2021; Sánchez‐Cuervo et al., 2012).

Our spatio‐temporal approach provides empirical evidence to this *Δ*TC potential at continental scales, as well as proves that the ecosystem change leading to a regime shift—in the context of both tree cover loss and gain—is indeed intensified at intermediate tree cover (Hirota et al., 2011) (Figure 2). This change in tree cover structure (*Δ*TC) across different $\bar{P}$ levels, thus, agrees with our spatio‐temporal hypothesis (Figures 1a and 2). Furthermore, spatially mapping these stable and unstable states provides us with key regions where forest conservation and management efforts need to be strengthened.

### 3.2 Forest stability and adaptation dynamics

**FIGURE 3:**
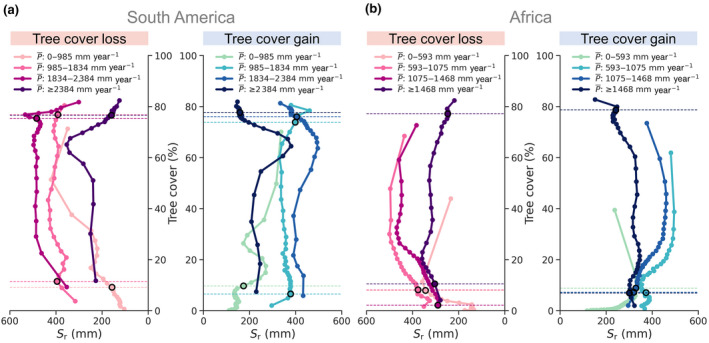
Relationship between mean precipitation ($\bar{P}$) and root zone storage capacity (*S*_r_) for (a) South America and (b) Africa. The solid lines correspond to median *S*_r_ for the bins of tree cover loss (left) and gain (right) in Figure 2. The points on the solid lines represent the centre of the individual bins. The (horizontal) dashed lines correspond to the minimum and maximum extent of the stable‐high and ‐low tree cover ecosystems, respectively, as defined in Figure 2a,c

But why can forest ecosystems maintain stability at different $\bar{P}$ levels and how does that relate to *Δ*TC (Figure 2)? The results from our *S*_r_ analysis suggest that forest ecosystems maintain their tree cover structure at decreasing $\bar{P}$ by increasing investment in their subsoil structure (Figure 3). Here, we observe a steep increase in *S*_r_ with both decreasing $\bar{P}$ and tree cover (Figure 3). In South America, within the stability extent of tree cover from 85% to 75%, the *S*_r_ increases up to 600 mm with decreasing $\bar{P}$ (Figure 3a). For Africa, although only a small portion of the forest is in this comparatively low *S*_r_ high‐tree cover stable state, we still observe a steep increase in *S*_r_ near the stable‐high tree cover state with decreasing $\bar{P}$ (Figure 3b). The least *Δ*TC within this stability extent reveals that stabilising feedbacks within the stable‐high tree cover (forest) ecosystems’ respond to the change in $\bar{P}$ by instigating *S*_r_ investment (Figures 2 and 3). This *S*_r_ investment, in reality, is the vertical and lateral growth of roots, allowing for more subsoil moisture storage. This subsoil storage thereby assists the forest ecosystems in maintaining their (stable) dense tree cover structure even under hydroclimatic stresses (Schenk & Jackson, 2002; Singh et al., 2020). However, this stabilising feedback of *S*_r_ investment to keep the ecosystems in a stable‐high tree cover state starts to change as we move to the intermediate tree cover.

At (unstable) intermediate tree cover, we find *Δ*TC to gradually increase and maximise around 40%–50% tree cover (Figure 2). We also find that the steep increase in *S*_r_ gradually maximises around the 70%–60% tree cover and remains unchanged between 60% and 30% tree cover (Figure 3), thus suggesting causation between maximum *S*_r_ investment and changes to ecosystem structure. When analysing the changes to the forest ecosystems’ structure against varying levels of drought and fire stress at the local scale (Figures S4 and S5), we observe that unstable state forests—in comparison to stable‐high tree cover ecosystems—have often maximised their *S*_r_ investment and show deterioration to a savannah‐like state.

These deteriorations are not sudden but gradual over time. This suggests that there exists a certain maximum investment potential beyond which the shift from forest to a savannah state becomes eminent (Singh et al., 2020), which manifests itself as relatively high rate of *Δ*TC over time for the unstable forest ecosystems (Figure 2; Figures S4 and S5). Considering *S*_r_ along with $\bar{P}$, therefore, has allowed us to evaluate the invisible buffering responses of forest ecosystems which otherwise are not apparent but are critical to the stability of the forest ecosystems. Overall, these responses are specifically catered towards efficiently optimising the available water resources and modifying the ecosystem's aboveground tree cover structure (Migliavacca et al., 2021), and thus is able to manifest the shifts between the transient (stable and unstable) states as different magnitudes of *Δ*TC.

### 3.3 Resilience of the rainforest

**FIGURE 4:**
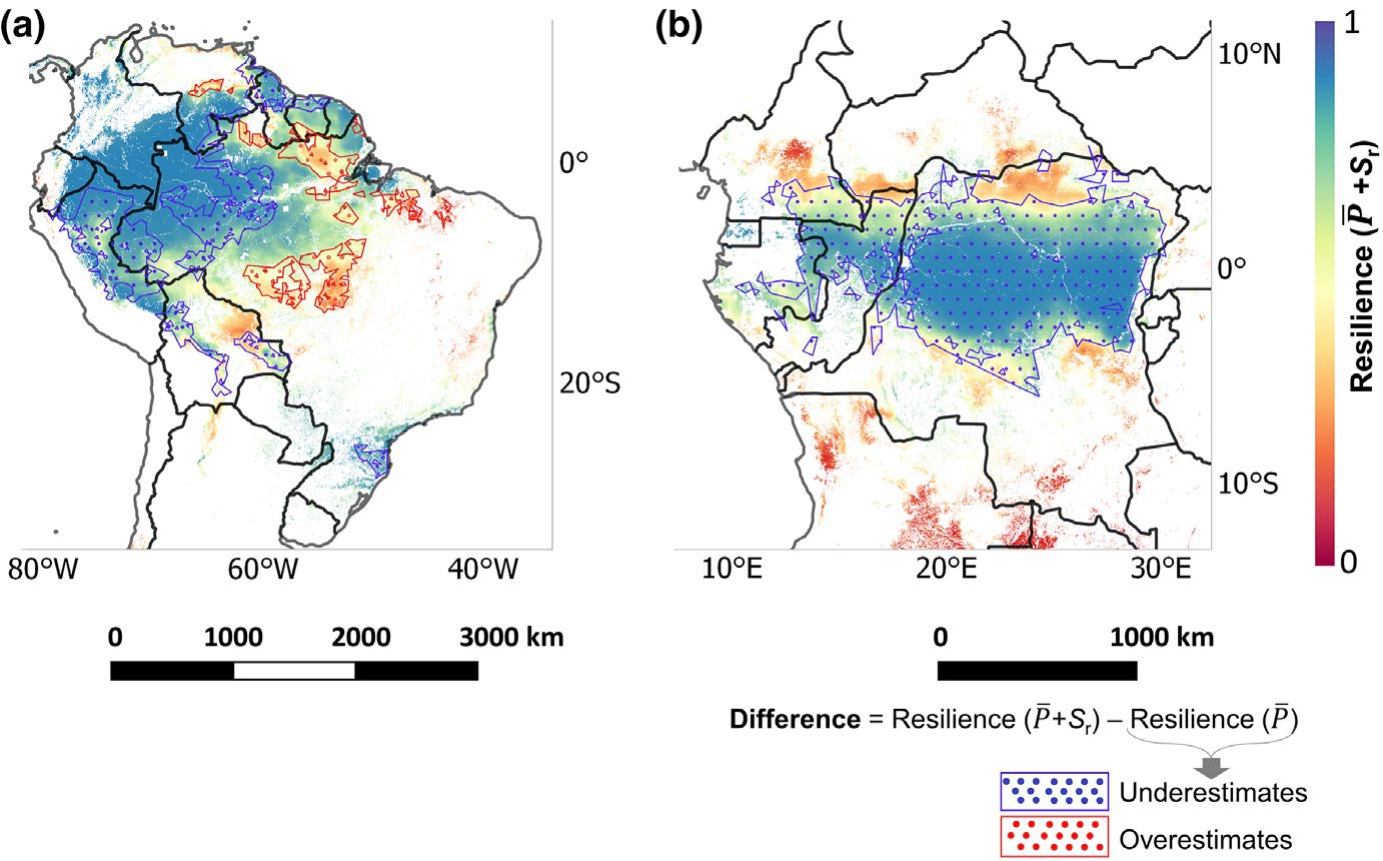
Resilience of the rainforest ecosystem. These resilience estimates are derived using the logistic regression based on $\bar{P}$ and *S*_r_ for both (a) South America and (b) Africa. Here, a value of ‘1’ implies a forest ecosystem with the highest resilience, and ‘0’ implies a forest ecosystem with the lowest resilience. Comparing the two resilience metrics, we observed that by considering only $\bar{P}$ (in resilience calculation), the resilience estimates show considerable differences for the Amazon and Congo rainforests (exact difference in Figure S6). Regions with tree cover ≤50% and human‐influenced land use (see Section 2) are masked

This study quantifies resilience using logistic regression that predicts the probability of the occurrence of a forest ecosystem (tree cover >50%) as a function of both $\bar{P}$ and *S*_r_ for respective continents (Tables S1 and S2). It predicts resilience between a scale of 0 to 1, where 1 represents the highest probability of finding forest—interpreted as a highly resilient forest ecosystem against perturbations—given the $\bar{P}$ and *S*_r_ estimates in the recent decades. Figure 4 shows that the most resilient forests are located in the central and central‐western parts of Amazon rainforests in South America, and a major portion of the central Congo rainforests in Africa. At the same time, the least resilient forests are in the central‐eastern and southern corridor of the Amazon rainforest (along the ‘Amazonian arc of deforestation’) and northern and southern parts of the Congo rainforest (Figure 4).

The $\bar{P}+S_{r}$‐based resilience metric shows that the resilience of a large portion of the Congo rainforest is higher than previously presumed (based on $\bar{P}$ only) (Hirota et al., 2011), whereas the resilience of Amazon rainforests shows minor differences (Figure 4; Figure S6) (Staal et al., 2018). Due to the unique evolutionary history of their respective ecology and climatology (Morley, 2000), high wet‐season precipitation has allowed for Amazonian rainforests species to have larger subsoil storage (i.e. *S*_r_) to buffer the water deficit than the Congo rainforests (Guan et al., 2015; Zhou et al., 2014). The grass species in Congo rainforests, on the contrary, have evolved to be highly water‐efficient (Still et al., 2003). This reduces the competitiveness between trees and grasses for moisture uptake (Singh et al., 2020), thereby increasing the resilience of the overall Congo rainforest ecosystem, even with low *S*_r_, against water deficit. Therefore, including *S*_r_ in our resilience metric has allowed us to capture this grass species‐induced drought coping strategy in Congo rainforests, which otherwise is hard to detect with just $\bar{P}$. Nevertheless, the resilience of both these rainforest ecosystems are declining due to increasing regional climatic risks (Phillips et al., 2009) and combined feedbacks from local deforestation and human‐induced fires (Davidson et al., 2012; Malhi et al., 2008).

**FIGURE 5:**
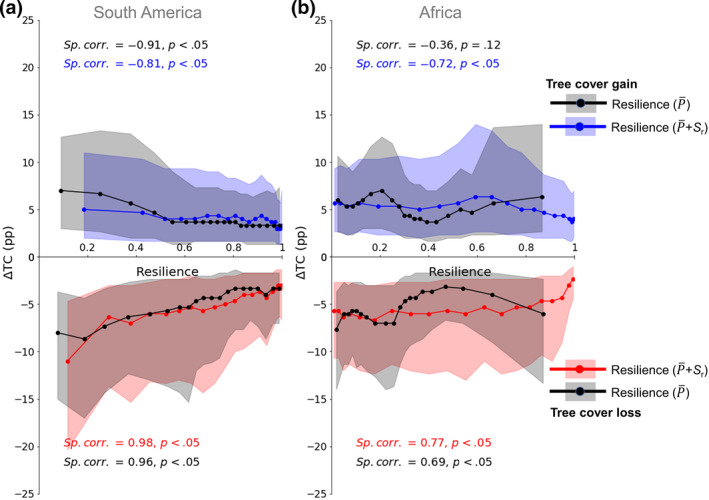
Validating the resilience estimates of the rainforest ecosystem with actual tree cover change (*Δ*TC) for (a) South America and (b) Africa. The samples are divided into 20 equally weighted intervals (similar to Figure 2). The dots in blue (i.e., *Δ*TC ≥ 0) and red (i.e., *Δ*TC < 0) correspond to our $\bar{P}$ and *S*_r_) resilience metric, whereas dots in black correspond to $\bar{P}$‐derived resilience estimates. The shaded regions represent the first and third quantile. The statistical test calculates the Spearman rank correlation (Sp. corr.) coefficient with an associated *p*‐value

Validation with actual *Δ*TC shows that the $\bar{P}+S_{r}$‐based resilience estimates perform better than only the $\bar{P}$‐based resilience (Figure 5). The performance of these resilience metrics based on *Δ*TC further strengthens our original hypothesis that more resilient ecosystems will tend to have lower *Δ*TC and vice versa (Figures 1a and 5). Although $\bar{P}$ is an important variable defining the broad influence of moisture on the ecohydrology of the ecosystem, considering *S*_r_ accounts for the local‐scale ecosystem adaptation of forests to buffer and withstand hydroclimatic changes (Singh et al., 2020), and is thus able to better represent the resilience of the rainforest ecosystems (Table S1). This better representation of ecosystem resilience can play a crucial role in management and conservation efforts (Newton, 2016).

## 4 CONCLUSIONS

We demonstrate that our observation‐based spatio‐temporal approach, which analyses ΔTC and *S*_r_ over the last two decades, provides empirical evidence of alternative stable states in the tropical terrestrial ecosystem of South America and Africa. We observe that the stable ecosystems at >75% and <10% tree cover show low ΔTC by instigating higher *S*_r_ investment. For stable ecosystems, *S*_r_ investment does not come at the expense of changes in aboveground forest structure. Compared to stable ecosystems, unstable ecosystems show much high *Δ*TC manifesting at intermediate tree cover of 30%–60% due to the inability of the ecosystems to utilise a similar level of investment. These tree cover ranges of stability and instability resemble the stability landscape of the previous space‐for‐time substitution‐based approach.

By only considering $\bar{P}$, the resilience of the ecosystems can be underestimated, which we observe for a considerable portion in the Congo rainforests. Only by modifying the existing, commonly used $\bar{P}$‐based resilience metric with an extended $\bar{P}+S_{r}$ metric, we account for both the influence of hydroclimate (i.e. $\bar{P}$) and the hydroclimatic adaptive capacity of the ecosystem (i.e. *S*_r_). Furthermore, the $\bar{P}+S_{r}$ resilience metric shows better performance and consistency with actual *Δ*TC, thus strengthening its performance over the $\bar{P}$‐based metric. Overall, this study accounts for the ecosystems temporal and adaptation dynamics which are becoming increasingly important to assess the transient state of the ecosystems under rapidly changing hydroclimatic conditions.

## CONFLICT OF INTEREST

The authors declare no competing interests.

## Supporting information

Supplementary Material

## Data Availability

The python and google earth engine code used for the analyses presented in this study is available from GitHub: https://github.com/chandrakant6492/Ecosystems‐stability‐and‐resilience. The python code for calculating root zone storage capacity is also available from GitHub: https://github.com/chandrakant6492/Drought‐coping‐strategy. The resilience maps generated for this study are available at Zenodo: https://doi.org/10.5281/zenodo.5878792. Other publicly available datasets that support the findings of this study are available at: (P‐CHIRPS) https://data.chc.ucsb.edu/products/CHIRPS‐2.0/, (E‐BESS) ftp://147.46.64.183/, (E‐FLUXCOM) ftp.bgc‐jena.mpg.de, (E‐PML) https://data.csiro.au/collections/#collection/CIcsiro:17375v2, (MOD44B_v6) https://lpdaac.usgs.gov/products/mod44bv006/, (Globcover) http://due.esrin.esa.int/page_globcover.php, (SPEI) https://spei.csic.es/database.html, (FireCCI51) https://geogra.uah.es/fire_cci/firecci51.php
