# Supplementary material for: Hydroclimatic adaptation critical to the resilience of tropical forests
Source: Glob Chang Biol. 2022 Feb 20;28(9):2930–9. doi: 10.1111/gcb.16115 (PMC9306811; doi:10.1111/gcb.16115)
Supplement: Supplementary file 1 — Supplementary Material [file GCB-28-2930-s001.docx]

**Supplementary Information**

**Hydroclimatic adaptation critical to the resilience of tropical forests**

**Chandrakant Singh^1,2^,*, Ruud van der Ent^3,4^, Lan Wang-Erlandsson^1,2^, Ingo Fetzer^1,2^**

**Contents**

[Supplementary Method-1: Root zone storage capacity calculation 2](#_Toc86161257)

[Supplementary Method-2: Perturbation trends 3](#_Toc86161258)

[Supplementary Method-3: Forest resilience calculation 3](#_Toc86161259)

[Supplementary Tables 5](#_Toc86161260)

[**Supplementary Table 1 |** Performance of logistic regression models considered for determining the resilience of the tropical ecosystem 5](#_Toc86161261)

[**Supplementary Table 2 |** Parameters of the logistic regression that predicts the probability of tree cover as a function of $\overline{P}$ and *S*_r_.. 5](#_Toc86161262)

[Supplementary Figures 6](#_Toc86161263)

[**Supplementary Fig. 1** | Tree cover change (*Δ*TC) distribution for South America. 6](#_Toc86161264)

[**Supplementary Fig. 2 |** Spatial distribution of **(a)** mean tree cover (from 2000-2019; %), **(b)** mean precipitation ($\overline{P}$ from 2000-2019; mm yr^-1^), **(c)** root zone storage capacity (S*_r_* from 2001-2012; mm), and **(d)** land cover. 7](#_Toc86161265)

[**Supplementary Fig. 3 |** We observe some exceptions in South America where ecosystems do not follow the hypothesised trend of *Δ*TC (Fig. 1a and 2). 8](#_Toc86161266)

[**Supplementary Fig. 4 |** Influence of climate and fire in influencing the tree cover of the ecosystem at different regions of interest (ROI) for South America. 9](#_Toc86161267)

[**Supplementary Fig. 5 |** Similar to Supplementary Fig. 4, the influence of climate and fire in influencing the tree cover of the ecosystem at different regions of interest (ROI) for Africa. 10](#_Toc86161268)

[**Supplementary Fig. 6 |** Resilience of the ecosystem derived from precipitation ($\overline{P}$; see Supplementary Table 1) for **(a)** South America and **(b)** Africa. 11](#_Toc86161269)

[Supplementary References 12](#_Toc86161270)

## Supplementary Method-1: Root zone storage capacity calculation

We have adopted root zone storage capacity (*S*_r_) from Singh et al. (2020). For this, we first calculated the water deficit ($D(t)$), which is based on daily accumulated water stress due to variable precipitation ($P(t)$) and evaporation ($E(t)$):

$D(t) = E(t)-P(t)$ (1)

where *t* denotes day count since the start of the simulation. The simulation for each grid cell starts in the month with the highest mean monthly precipitation (2001-2012) and runs for a whole year. The accumulated deficit $D_{a}(t+1)$ was integrated at each one-day timestep for one year such that it is either equal to or more than the deficit of the previous timestep, but never less than zero (as excess precipitation is assumed to run off as streamflow or groundwater recharge) using:

$D_{a}(t+1)=\max\{0,D(t)+D(t+1)\}$ (2)

Since the analysis assumes that vegetation adapts and responds to the critical dry period (Wang-Erlandsson et al., 2016), we compute the largest accumulated deficit per year $D_{a,y}$ by:

$D_{a,y}=\max\{D_{a}(t+1)\}\text{ }t=1:n-1$ (3)

where *n* equals the number of days in year *y*. Since this simulation is run for a whole year using precipitation and evaporation estimates, this mass-balance methodology does consider actual seasonal dynamics of precipitation (incoming moisture flux) and evaporation (outgoing moisture fluxes considering evaporation from soil moisture, interception, transpiration and open water (see methods) at diurnal timescale.

To avoid artificially introduced transitions between different biomes, a uniform 20-year drought return period based on the Gumbel extreme value distribution (Gumbel, 1958) was used to normalise all $D_{a,y}$. The Gumbel distribution ($F(x)$) is given by:

$F(x)=\exp\left[-\exp\left[-\frac{(x-\mu)}{\alpha}\right]\right]$ (4)

Where *μ* and *α* are the location and scale parameter, respectively. The python package ‘skextremes’ (Correoso, 2019) was used to calculate *S*_r_:

$S_{\text{r}}=D_{a,y}+K\times\sigma_{n-1}$ (5)

Where *K* is the frequency factor given by:

$K=\frac{y_{t}-y_{n}}{S_{n}}$ (6)

And *y_t_* is the reduced variate given by:

$y_{t}=-\left[\ln\left[\ln\left(\frac{T}{T-1}\right)\right]\right]$ (7)

Where *T* is the drought return period (i.e., 20 years in this study), $D_{a,y}$ is the mean annual accumulated deficit for the years 2001-2012, $\sigma_{n-1}$ is the standard deviation of the sample. Also, $y_{n}$ is the reduced mean and *S_n_* is the reduced standard deviation, which for *n* = 11 years equal to 0.4996 and 0.9676, respectively (Gumbel, 1958).

## Supplementary Method-2: Perturbation trends

Ecosystems lose their structural integrity due to both climatological and non-climatological factors (i.e., human-influenced). In this study, we only focus on environmental changes that are detectable using remotely sensed datasets. To analyse this, we considered two variables (i) fire, and (ii) drought severity. Fire modifications to the ecosystems were analysed using a global time series of burned areas (named FireCCI51; km^2^) derived from Moderate Resolution Imaging Spectroradiometer (MODIS). The data was procured for the years 2001-2019 at a resolution of 250 m x 250 m (Lizundia-Loiola et al., 2020). ESA Globcover dataset was used to remove pixels with human land use and non-terrestrial land cover from FireCCI51 dataset.

To analyse the drought severity, we used standardised precipitation evaporation index (SPEI) (Vicente-Serrano et al., 2013). SPEI is the modified extension of the standardised precipitation index. It is derived from climate-based datasets and has been widely used to determine the influence of droughts with varying magnitude and duration on the natural and human-influenced systems (Anderegg et al., 2020). SPEI integrates both rainfall (i.e., supply) and potential evaporation (i.e., demand) to capture climate trends (positive for wet and negative for dry climate). For our analysis, we experimented with different SPEI potential evaporation equations (Penman-Monteith (Allen et al., 1998) and Thornthwaite (Thornthwaite, 1948)). However, we did not observe any significant differences between the general SPEI trends. For our study, we used present the SPEI-12 (i.e. 12-month rolling average SPEI) data derived from the Climatic Research Unit (CRU) dataset, where potential evaporation was based on the Penman-Monteith equation. The SPEI-12 data was directly procured for the years 2000-2018 at 0.5° latitude $\times$ 0.5° longitude resolution. SPEI-12 algorithm aggregates precipitation and potential evaporation over a period of 12 months, and is thus convenient for evaluating annual (consistent with tree cover dataset) implications of droughts.

## Supplementary Method-3: Forest resilience calculation

The resilience metric is based on logistic regression adapted from Hirota et al. (2011), however, we also included *S*_r_ along with $\overline{P}$. We used the python package ‘statsmodel’ (Seabold & Perktold, 2010) for our analysis. The logistic regression predicting the resilience (*f(z)*) of the rainforest ecosystem was given by:

$f(z)=\frac{1}{1+\exp(-z)}$ (8)

$z=a+b(\overline{P})+c(S_{\text{r}})$ (9)

Where *z* = 1 when forest (i.e., tree cover > 50%), and *z* = 0 when savannah (i.e., tree cover ≤ 50%; including grasslands and treeless state). Also, $\overline{P}$ represents mean annual precipitation, and *S*_r_ represents root zone storage capacity.

## Supplementary Tables

### **Supplementary Table 1 |** Performance of logistic regression models considered for determining the resilience of the tropical ecosystem. The models are evaluated using (shown in increasing order of) the Akaike Information Criterion (AIC) and Bayesian Information Criterion (BIC). The model with least AIC and BIC is used in this study (Fig. 4).

|  | **South America** | | **Africa** | |
| --- | --- | --- | --- | --- |
| **Variables** | **AIC** | **BIC** | **AIC** | **BIC** |
| $\overline{P}$*+ S*_r_ | 501772.33 | 501806.75 | 249156.25 | 249191.01 |
| *S*_r_ | 582170.43 | 582193.38 | 257513.09 | 257536.26 |
| $\overline{P}$ | 638263.45 | 638286.40 | 454887.59 | 454910.76 |

### **Supplementary Table 2 |** Parameters of the logistic regression that predicts the probability of tree cover as a function of $\overline{P}$ and *S*_r_. Tree cover > 50% is considered as a high tree cover ecosystem, and tree cover ≤ 50% is considered as a low tree cover ecosystem. All parameters are statistically significant (p-value < 0.05).

| **Coefficient** | **South America** | **Africa** |
| --- | --- | --- |
| (Intercept) **a** | 0.7471 | 3.7822 |
| (for $\overline{P}$) **b** | 0.0015 | 0.0012 |
| (for *S*_r_) **c** | -0.0067 | -0.0162 |

## Supplementary Figures

**
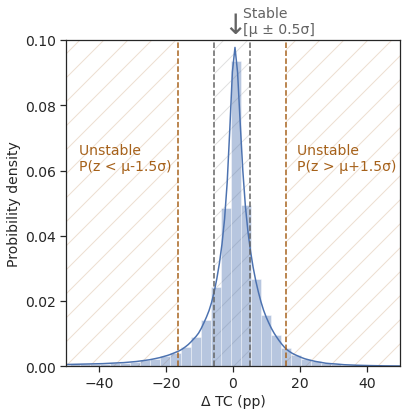
**

### **Supplementary Fig. 1** | Tree cover change (*Δ*TC) distribution for South America. The portion within the grey and outside the brown lines represents the stable and unstable regions, respectively. Here, *P(z)* refers to the probability distribution of the curve, μ is the mean (i.e., -0.26), and σ is the standard deviation (i.e., 10.81) of the distribution. *Δ*TC for Africa shows a similar trend, however, is not shown in this study.

**
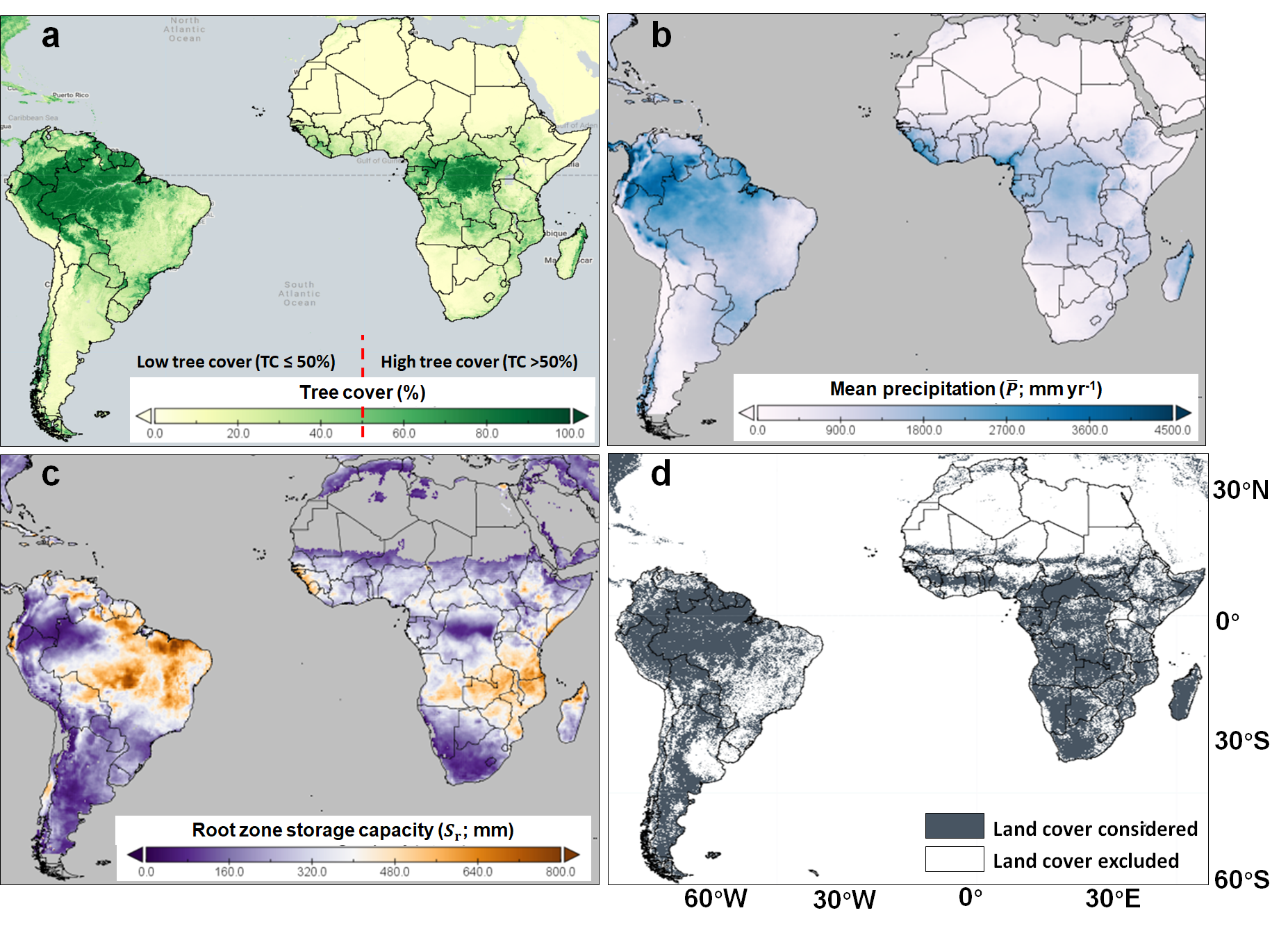
**

### **Supplementary Fig. 2 |** Spatial distribution of **(a)** mean tree cover (from 2000-2019; %), **(b)** mean precipitation ($\overline{P}$ from 2000-2019; mm yr^-1^), **(c)** root zone storage capacity (S*_r_* from 2001-2012; mm), and **(d)** land cover.

**
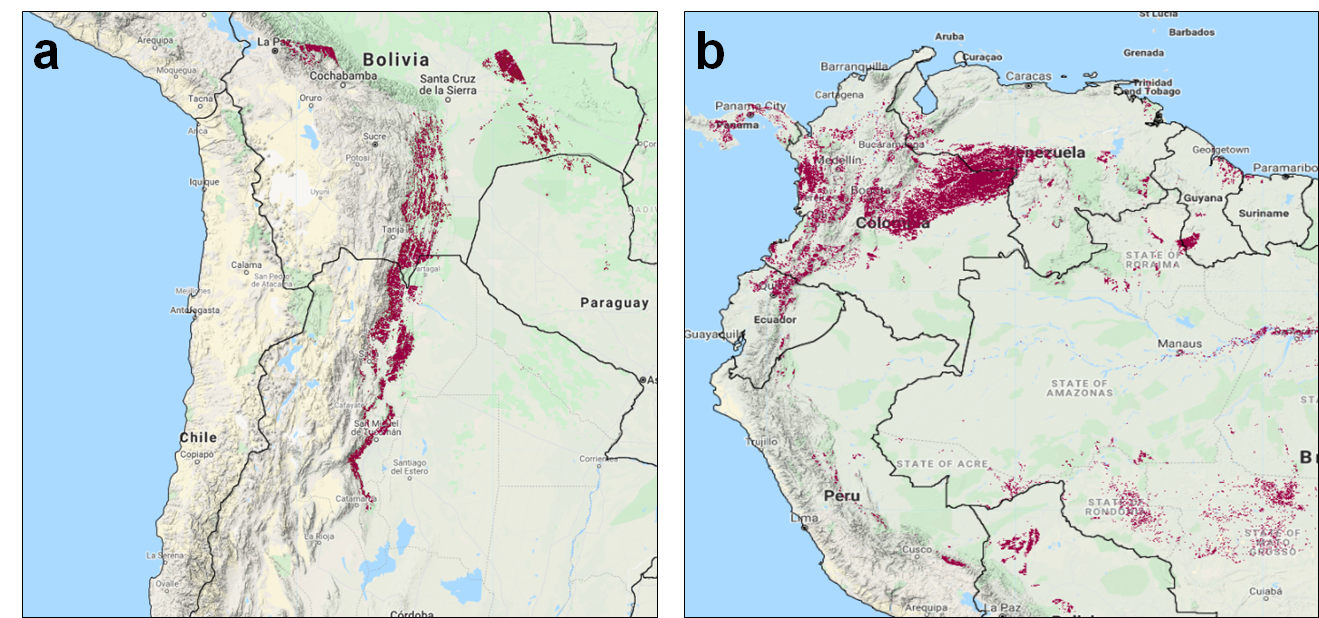
**

### **Supplementary Fig. 3 |** We observe some exceptions in South America where ecosystems do not follow the hypothesised trend of *Δ*TC (Fig. 1a and 2). We observe that the **(a)** high tree cover ecosystems (not-stable; tree cover > 65% at $\overline{P}$ < 985 mm yr^-1^ in Fig. 2a) at the foot of the Andes (marked in red) receives not only by local $\overline{P}$*,* but also gets substantial moisture from glacial runoff (Bradley et al., 2006; Herzog et al., 2011). These ecosystems are, therefore, able to sustain themselves even at low precipitation. **(b)** On the other hand, low tree cover ecosystems (not-stable; tree cover < 40% at $\overline{P}$ > 1,834 mm yr^-1^ in Fig. 2a) between (lowland) Colombia and Venezuela (marked in red) suffers from low nutrient soil characteristics, rapid leaching and an extreme precipitation seasonality, thus promoting a savannah ecosystem even at high precipitation (Blydenstein, 1967; Sarmiento, G., 1983).

**
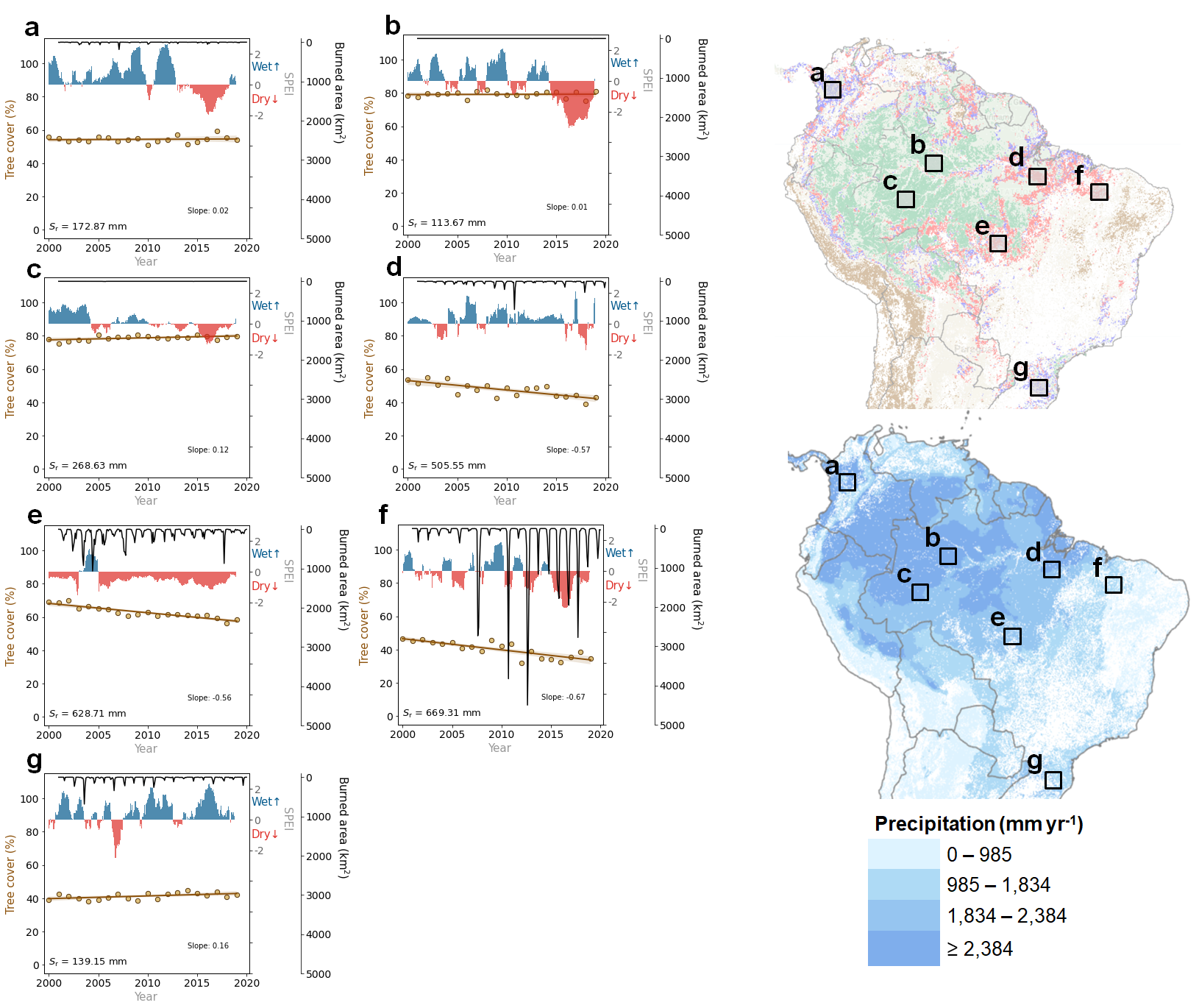
**

### **Supplementary Fig. 4 |** Influence of climate and fire in influencing the tree cover of the ecosystem at different regions of interest (ROI) for South America. The regression corresponds to the changes in mean annual tree cover values in the respective ROI. The shade around the regression line corresponds to the 95% confidence interval. Here, near zero slope values signify no tree cover change, positive slopes signify a tree cover gain, and negative slopes signify a tree cover loss over time. The 12-month standardised precipitation and evaporation index (SPEI) signifies progress of the wet (positive; blue) and dry (negative; red) climate in the region. The black lines ‒ on the top ‒ evaluates the total burnt area due to the influence of fire in the respective ROI.


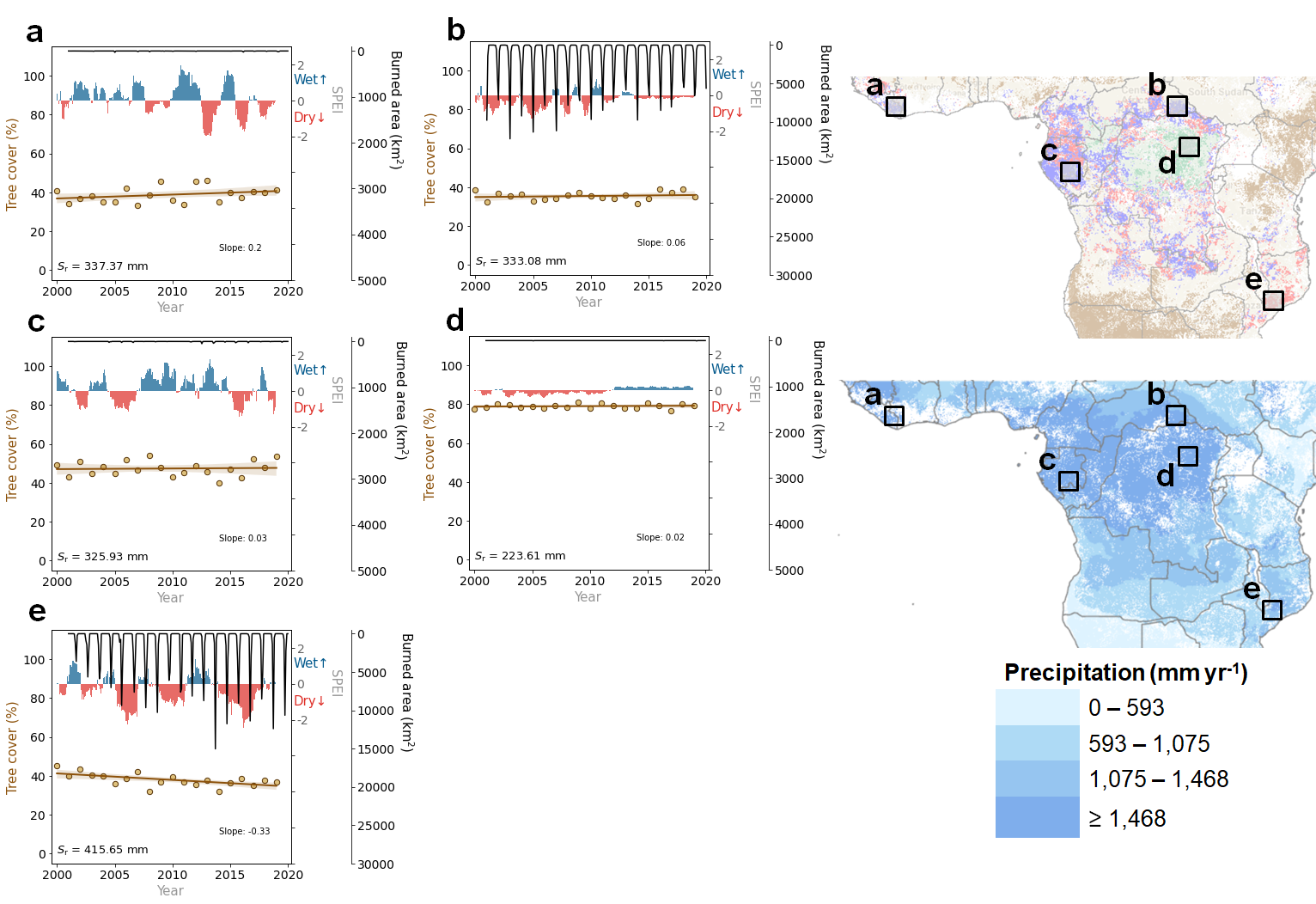


### **Supplementary Fig. 5 |** Similar to Supplementary Fig. 4, the influence of climate and fire in influencing the tree cover of the ecosystem at different regions of interest (ROI) for Africa.


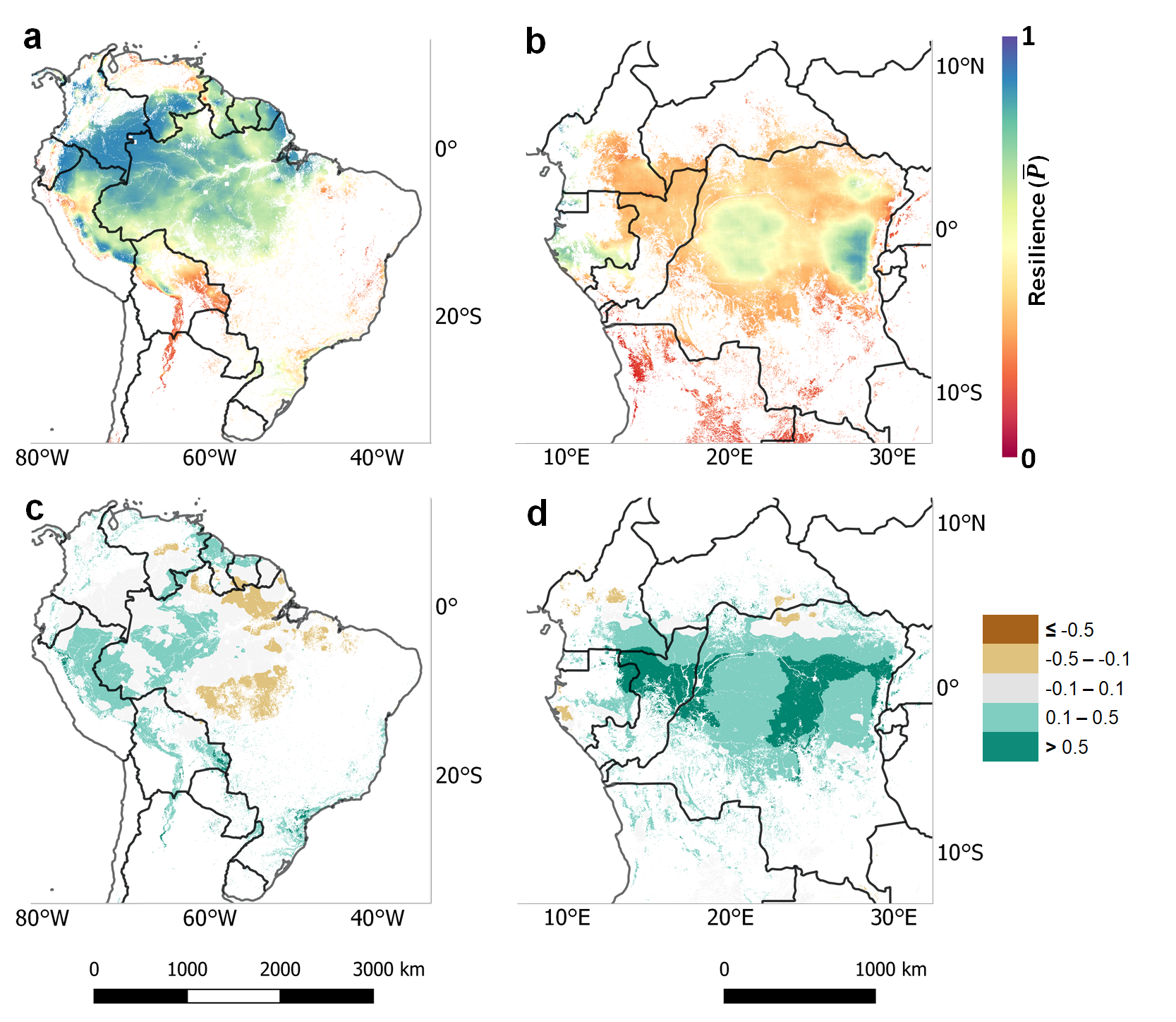


### **Supplementary Fig. 6 |** Resilience of the ecosystem derived from precipitation ($\overline{P}$; see Supplementary Table 1) for **(a)** South America and **(b)** Africa. The spatial extent only shows the resilience for regions with tree cover > 50%. **(c, d)** Difference between the resilience used in this study ($\overline{P}$+*S*_r_ in Fig. 4; Supplementary Table 1) and only $\overline{P}$-derived resilience metric (a, b).

## Supplementary References

Allen, G. A., Pereira, L. S., Raes, D., & Smith, M. (1998). *Crop Evapotranspiration: Guidelines for computing crop water requirements*. Food and Agriculture Organization of the United Nations, Rome. https://agris.fao.org/agris-search/search.do?recordID=SO2005100020

Anderegg, W. R. L., Trugman, A. T., Badgley, G., Konings, A. G., & Shaw, J. (2020). Divergent forest sensitivity to repeated extreme droughts. *Nature Climate Change*, *10*(12), 1091–1095. https://doi.org/10.1038/s41558-020-00919-1

Blydenstein, J. (1967). Tropical Savanna Vegetation of the Llanos of Colombia. *Ecology*, *48*(1), 1–15. https://doi.org/10.2307/1933412

Bradley, R. S., Vuille, M., Diaz, H. F., & Vergara, W. (2006). Threats to Water Supplies in the Tropical Andes. *Science*, *312*(5781), 1755–1756. https://doi.org/10.1126/science.1128087

Correoso, K. (2019, September 27). *Skextremes Documentation*. https://github.com/kikocorreoso/scikit-extremes

Gumbel, E. J. (1958). *Statistics of extremes.* Columbia University Press.

Herzog, S., Martinez, R., Jørgensen, P., & Tiessen, H. (2011). *Climate change and biodiversity in the tropical Andes*. Inter-American Institute for Global Change Research (IAI) and Scientific Committee on Problems of the Environment (SCOPE). https://doi.org/10.13140/2.1.3718.4969

Hirota, M., Holmgren, M., Van Nes, E. H., & Scheffer, M. (2011). Global Resilience of Tropical Forest and Savanna to Critical Transitions. *Science*, *334*(6053), 232–235. https://doi.org/10.1126/science.1210657

Lizundia-Loiola, J., Otón, G., Ramo, R., & Chuvieco, E. (2020). A spatio-temporal active-fire clustering approach for global burned area mapping at 250 m from MODIS data. *Remote Sensing of Environment*, *236*, 111493. https://doi.org/10.1016/j.rse.2019.111493

Sarmiento, G. (1983). *The Savannas of tropical America*. Elsevier, Amsterdam, The Netherlands.

Seabold, S., & Perktold, J. (2010). *Statsmodels: Econometric and Statistical Modeling with Python*. 92–96. https://doi.org/10.25080/Majora-92bf1922-011

Singh, C., Wang-Erlandsson, L., Fetzer, I., Rockström, J., & Ent, R. van der. (2020). Rootzone storage capacity reveals drought coping strategies along rainforest-savanna transitions. *Environmental Research Letters*, *15*(12), 124021. https://doi.org/10.1088/1748-9326/abc377

Thornthwaite, C. W. (1948). An Approach toward a Rational Classification of Climate. *Geographical Review*, *38*(1), 55–94. https://doi.org/10.2307/210739

Vicente-Serrano, S. M., Gouveia, C., Camarero, J. J., Beguería, S., Trigo, R., López-Moreno, J. I., Azorín-Molina, C., Pasho, E., Lorenzo-Lacruz, J., Revuelto, J., Morán-Tejeda, E., & Sanchez-Lorenzo, A. (2013). Response of vegetation to drought time-scales across global land biomes. *Proceedings of the National Academy of Sciences*, *110*(1), 52–57. https://doi.org/10.1073/pnas.1207068110

Wang-Erlandsson, L., Bastiaanssen, W. G. M., Gao, H., Jägermeyr, J., Senay, G. B., van Dijk, A. I. J. M., Guerschman, J. P., Keys, P. W., Gordon, L. J., & Savenije, H. H. G. (2016). Global root zone storage capacity from satellite-based evaporation. *Hydrology and Earth System Sciences*, *20*(4), 1459–1481. https://doi.org/10.5194/hess-20-1459-2016
